# Supplementary material for: Loss of microRNA-135b Enhances Bone Metastasis in Prostate Cancer and Predicts Aggressiveness in Human Prostate Samples
Source: Cancers (Basel). 2021 Dec 9;13(24):6202. doi: 10.3390/cancers13246202 (PMC8699528; doi:10.3390/cancers13246202)
Supplement: Supplementary file 1 [file cancers-13-06202-s001.zip › Figure S1_cBioportal .pdf]

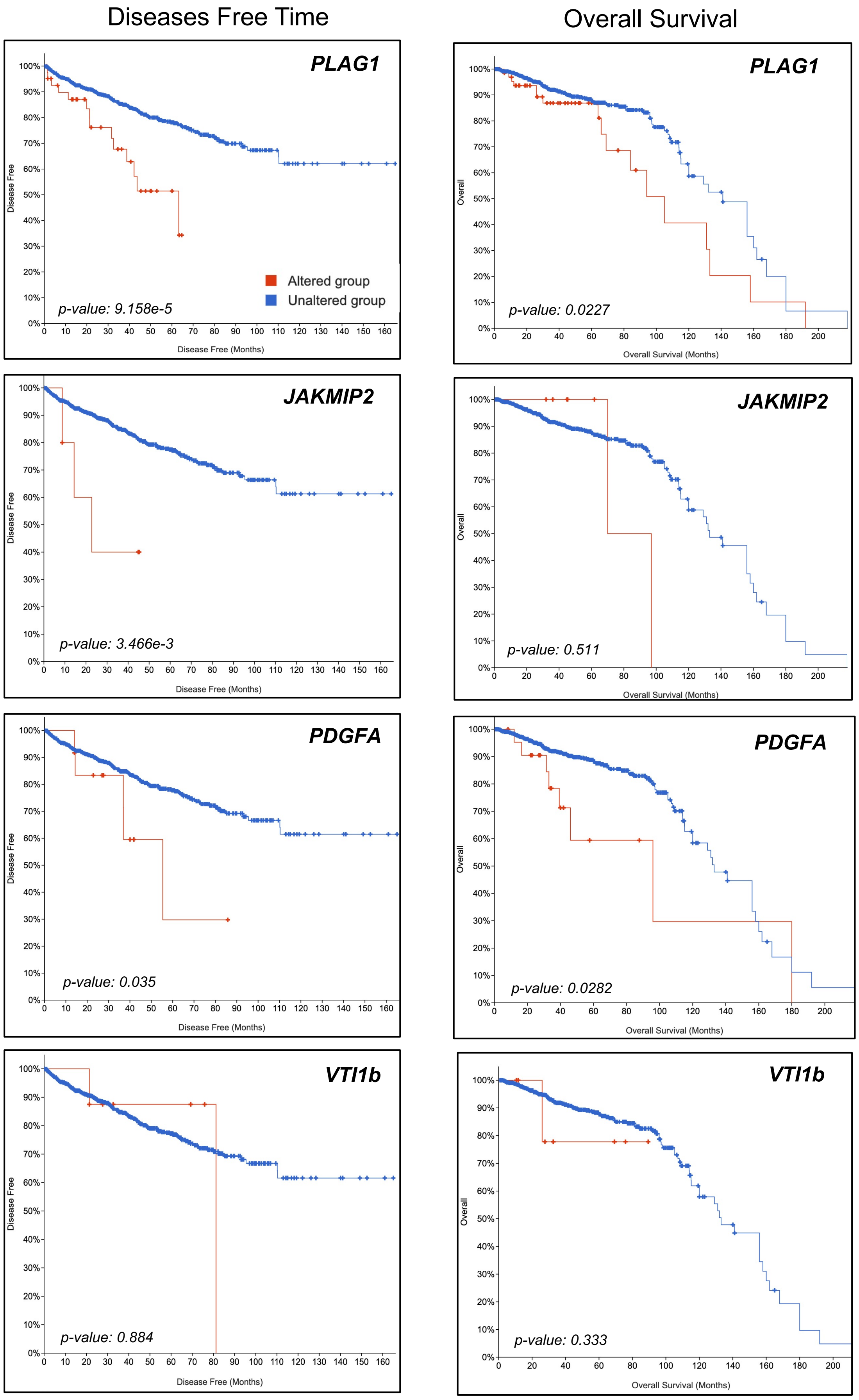

Figure S1. Correlation between diseases free time (in months) and overall survival with the expression levels of miR-135b validated targets. Patients' data were retrieved from different prostate cancer studies and TCGA Repository using the cBioportal Visualization platform.
